# Manual of Eye-Surgery

**Published:** 1874-06

**Authors:** 


### BIBLIOGRAPHICAL.

Manual of Eye-Surgery. By A. J. Howe, A.M., M.D. Author of "A Treatise on Fractions and Dislocations," etc. Cincinnati: Printed by Wilstach, Baldwin & Co. 1874.

This is an excellent manual by a gentleman who understands practically whereof he teaches. It will fill a place, well and conveniently, in the libraries of the active practitioner, who will find the practice good and the matter sufficiently condensed to meet his daily wants.

